# The oncofetal gene survivin is re-expressed in osteoarthritis and is required for chondrocyte proliferation *in vitro*

**DOI:** 10.1186/1471-2474-12-150

**Published:** 2011-07-05

**Authors:** Philipp Lechler, Sanjeevi Balakrishnan, Jens Schaumburger, Susanne Grässel, Clemens Baier, Joachim Grifka, Rainer H Straub, Tobias Renkawitz

**Affiliations:** 1 Department of Orthopedic Surgery, University of Regensburg, Asklepios Klinikum Bad Abbach, Kaiser Karl V Allee 3, 93077 Bad Abbach, Germany; 2 Division of Immunology and Inflammation (Medicine), Imperial College London, Hammersmith Hospital, Du Cane Road, London W12 0HS, UK; 3 Laboratory of Experimental Rheumatology and Neuroendocrino-Immunology, Division of Rheumatology, Department of Internal Medicine I, University Hospital Regensburg, Franz-Josef-Strauß-Allee 11, 93053 Regensburg, Germany; 4 Centre for Biomedical Technology, BioPark 1, Josef-Engert-Str. 9 93053 Regensburg, Germany

**Keywords:** apoptosis, chondrocyte, osteoarthritis, proliferation, survivin

## Abstract

**Background:**

Regulation of cell death and cell division are key processes during chondrogenesis and in cartilage homeostasis and pathology. The oncogene survivin is considered to be critical for the coordination of mitosis and maintenance of cell viability during embryonic development and in cancer, and is not detectable in most adult differentiated tissues and cells. We analyzed survivin expression in osteoarthritic cartilage and its function in primary human chondrocytes *in vitro*.

**Methods:**

Survivin expression was analyzed by immunoblotting and quantitative real-time PCR. The localization was visualized by immunofluorescence. Survivin functions *in vitro *were investigated by transfection of a specific siRNA.

**Results:**

Survivin was expressed in human osteoarthritic cartilage, but was not detectable in macroscopically and microscopically unaffected cartilage of osteoarthritic knee joints. In primary human chondrocyte cultures, survivin was localized to heterogeneous subcellular compartments. Suppression of survivin resulted in inhibition of cell cycle progression and sensitization toward apoptotic stimuli *in vitro*.

**Conclusions:**

The present study indicates a role for survivin in osteoarthritic cartilage and human chondrocytes. *In vitro *experiments indicated its involvement in cellular division and viability. Learning more about the functions of survivin in chondrocyte biology might further help toward understanding and modulating the complex processes of cartilage pathology and regeneration.

## Background

Endochondral ossification describes the formation of a cartilaginous skeleton and its subsequent replacement by mineralized bone. In the growth plate, complex processes regulate the highly ordered sequences of chondrocyte proliferation, differentiation and apoptosis, resulting in skeletal growth [1]. Even minor disturbances in this delicate balance lead to abnormalities of endochondral bone development, resulting in skeletal dysplasia. Proliferation of immature chondrocytes is stimulated by parathyroid hormone-related protein (PTHrP) and other factors [2], while mitotic activity in mature chondrocytes is a rare event and confined to pathologic scenarios including osteoarthritis. The molecular regulation of the progressive loss of proliferative capacity is still not completely understood and remains a major challenge for future therapeutic strategies. Regenerative approaches using autologous chondrocytes are further complicated by the limited life span of chondrocytes *in vitro *and their enhanced susceptibility to proapoptotic stressors [3]. Under physiologic conditions, programmed cell death in cartilage is uncommon owing to the maintenance of metabolic homeostasis and chondrocyte adhesion to extracellular matrix proteins [4,5]. In osteoarthritis, the influence of proapoptotic mechanical and metabolic factors increases and is antagonized by the initiation of various molecular antiapoptotic mechanisms [6-8]. The initiation of the various protective molecular mechanisms have been discussed in previous studies [9-11].

A protein believed to be involved in cellular division and prevention of cell death is survivin. At 16.8 kDa, survivin is the smallest member of the inhibitor of apoptosis gene family (IAP), and comprises one N-terminal baculovirus IAP repeat (BIR) domain and a long C-terminal-helix coiled region. The regulation of survivin involves transcriptional, translational and post-translational modifications [12]. Since its first description, survivin was thought to be confined to embryonic development and cancers and hardly expressed in adult differentiated tissues. Survivin is ubiquitously expressed in embryonic tissues, and homozygous knockout mice for survivin show embryonic lethality as early as day 4.5 postcoitum [13]. In adult organisms, survivin is highly re-expressed in solid tumors and malignant cells, as shown by a large body of evidence. Furthermore, correlations between survivin expression, tumor growth, aggressiveness and overall prognosis have been demonstrated convincingly [14-16]. Understandably, survivin has been proposed as a perfect molecular target for future oncologic therapies. However, recent studies questioned the oncofetal paradigm of survivin expression and reported a role of survivin in non-malignant tissues and normal cells [17]. A limited insight into the role of survivin in the musculoskeletal apparatus beyond the oncologic context has been gained through previous studies. In rheumatoid arthritis (RA), high levels of survivin mRNA and protein have been reported in the inflamed synovial membrane [18-21], synovial fluid [22,23] and peripheral blood samples [24]. Of note, survivin expression has been discussed as a reliable predictor of disease severity in RA [22,24].

In contrast to RA, the role of survivin in osteoarthritic joints has not been clarified. This study describes survivin expression in primary human chondrocytes *in vitro *and reports selective survivin re-expression in human osteoarthritic cartilage.

## Methods

Unless otherwise stated, all chemicals were purchased from Sigma-Aldrich (Taufkirchen, Germany).

### Collection of human tissues

Articular cartilage was collected from 20 patients with osteoarthritis undergoing total knee replacement. The mean patient age was 62.5 years (range, 45-75 years). The cartilage biopsies were fixed in 4% paraformaldehyde for immunohistochemistry and/or prepared for cell isolation (see below). Arthritic cartilage sections were classified as either osteoarthritic or non/moderate osteoarthritic cartilage specimens. For this purpose, three bunch biopsies (1.5 mm) were collected from three different areas of each cartilage specimen. After Safranin O staining, the specimens were analyzed for the degree of histological change [25]. Written informed consent was obtained from each patient before the arthroplasty. The collection of human tissues was approved by the local Ethics Committee (No. 09/131).

### Human primary chondrocytes and cell culture conditions

For cell culture studies, primary human chondrocytes were isolated as previously described [26]. The isolated chondrocytes were plated in 75-cm^2 ^flasks with medium comprising a 1:1 mixture of Dulbecco's modified Eagle's medium and Ham's F-12 supplemented with 10% fetal calf serum (PAA, Cölbe, Germany), and incubated at 37°C under 5% CO_2 _in humidified air. All experiments were conducted during passage 2 and subconfluent cultures were used. At passage 2, all established cultures expressed Sox9 and collagen type II, alpha 1 (COL2A1) mRNA as measured by real-time PCR (data not shown).

### Protein extraction and immunoblot analysis

Protein extraction of cultured cells was performed as previously described [27]. Briefly, for cell culture extracts, adherent cells were washed and removed by scraping, and centrifuged for 5 minutes at 750 rpm. Cell pellets were homogenized in extraction buffer (Roche Applied Science, Mannheim, Germany). For survivin immunoblotting, proteins were resolved by sodium dodecyl sulfate-polyacrylamide gel electrophoresis using 10% gels and blotted onto Immobilon P Membranes (Millipore, Bedford, MA). The membranes were blocked in 5% fat-free dried milk and probed with primary antibodies. After incubation with horseradish peroxidase-conjugated secondary antibodies, the positive bands were visualized by chemiluminescence (Pierce, Rockford, IL). The details of all the primary and secondary antibodies used are given in Table 1. As a control for antibody specificity, we loaded reticulocyte lysates programmed with a full-length human survivin cDNA (data not shown).

**Table 1:** Details of the antibodies used

Method	Detected	Primary antibody	(μg/ml)	secondary antibody	(μg/ml)
	protein				
IB	Survivin	pAB AF886 (R&D Systems)	1,0	Polyclonal immunoglobulins/HRP-conjugated (DAKO)	0,3
IB	Survivin	pAB 500.201 (Novus Biologicals)	1,0	Polyclonal immunoglobulins/HRP-conjugated (DAKO)	0,5
IF	Survivin	pAB AF886 (R&D Systems)	10,0	Red fluorescent dye-labeled immunoglobulin (Invitrogen)	10,0
IF	Survivin	pAB 500.201 (Novus Biologicals)	10,0	Red fluorescent dye-labeled immunoglobulin (Invitrogen)	8,0
IF	Survivin	mAB clone 60.11 (Novus Biologicals)	6,0	Red fluorescent dye-labeled immunoglobulin (Invitrogen)	8,0

The primary and secondary antibodies used for the immunofluorescence and immunoblotting are listed, with inclusion of the sources, purposes and concentrations. IB: immunoblotting; IF: immunofluorescence.

### Survivin immunofluorescence

Primary human chondrocytes cultured on glass slides or paraffin-embedded cartilage specimens were processed as follows. For antigen retrieval, the slides were boiled for 20 minutes (10 mM citrate buffer, pH 6.0). Nonspecific binding sites were blocked with 5% fat-free dried milk. The sections were incubated with primary antibodies for 12 hours at 4°C in a humidified chamber and incubated with red fluorescent dye-labeled anti-rabbit IgG. DNA was stained with 4,6-diamidino-2-phenylindole (DAPI). The slides were observed and photographed using a fluorescence microscope (Zeiss, Jena, Germany). To determine the antibody specificity and validity of the immunofluorescence, four independent antibodies were applied. The details of all the primary and secondary antibodies used are given in Table 1. After equal incubation times, omission of the primary or secondary antibody resulted in completely negative signals at comparable exposure times. As a positive control, paraffin-embedded specimens of human high-grade chondrosarcoma were used.

### RNA extraction and real-time PCR

Survivin mRNA expression was assayed by real-time PCR as previously described in detail [14]. RNA extraction was performed using an RNeasy micro kit (Qiagen, Hilden, Germany) according to the protocol originally described by McKenna et al. [28]. Total RNA (1 μg) was transcribed into cDNA using a Sensiscript RT kit (Qiagen). For real-time PCR, intron-spanning primer sequences for human survivin were applied. The controls used were human GAPDH (for primer details, see Table 2) and β-actin. All primers were used at a concentration of 300 nmol/L, with 55°C as the annealing temperature. A commercial 2 SYBR Green PCR Mix (Eurogentec, Seraing, Belgium) was used according to the manufacturer's instructions. PCR was performed with 50 cycles, taking 2 μl of cDNA into the reaction with an end volume of 25 μl. The values for survivin were related to their controls using the 2^-Δct ^calculation method.

**Table 2:** Details of the primer oligonucleotide sequences used for real-time PCR

Gene	Forward primer 5'-3'	Reverse primer 5'-3'
Survivin	CTTGGCCCAGTGTTTCTTCT	CCTCCCAAAGTGCTGGTATT
GAPDH	CCCACTCCTCCACCTTTGAC	CATACCAGGAAATGAGCTTGACAA
β-actin	AGTCCTGTGGCATCCACGAAA	GTCATACTCCTGCTTGCTGA
SOX9	CTGAGTCATTTGCAGTGTTTTCT	CATGCTTGCATTGTTTTTGTGT
COL2A1	CATTGATGGGGAGGCGTGAG	CATTGATGGGGAGGCGTGAG

### Survivin knockdown by siRNA

*For the transfection analysis, cells were seeded into 6-well dishes at 1.5 × 10^5 ^cells per 3.5-cm well at 24 hours before the knockdown was performed. *For knockdown of survivin, a short interfering RNA (siRNA) with the sequence of sense 5'-GCGCCUGCACCCCGGAGCG-3' and antisense 5'-CGCUCCGGGGUGCAGGCGC-3' was used as previously described [27]. A siRNA targeting green fluorescence protein (GFP) with the sequence of sense 5'-GUGUGCUGUUUGGAGGUCTT-3' and antisense 5'-GAACUCCAAACAGCACACCTT-3' was transfected as a negative control. All siRNAs were applied at a concentration of 100 nmol/L.

### Cell cycle analysis

Both adherent and detached cells were collected by trypsinization and resuspended in a staining solution containing 1.5 mol/L propidium iodide (PI) and 25 g/ml RNase A. The samples were subjected to fluorescence-activated cell sorting analysis (FACS) using a FACSCalibur (BD Biosciences, Heidelberg, Germany).

### Caspase 3/7 activity assay

Apoptosis was studied by measuring the activity of caspases 3 and 7 in a 96-well microplate format, using Caspase-Glo (Promega, Madison, WI). Chondrocytes were seeded at 1.5 × 10^5 ^cells per 3.5-cm well at 24 hours before the survivin-specific or control siRNA was transfected. For the analysis, the cells were incubated for 90 minutes in a luciferase substrate mix, and the luminescence activity was measured in a luminometer (Berthold, Bad Wildbad, Germany).

### Measurement of cell proliferation by quantification of BrdU incorporation

A commercial cell proliferation assay (Cell Proliferation ELISA; Roche Applied Science) was used according to the manufacturer's recommendations. Cells were cultured in a 96-well microtiter plate and exposed to BrdU for 4 hours. After application of a fixation solution, the cells were labeled with a peroxidase-conjugated mouse monoclonal antibody. Next, the bound antibody was quantified with a peroxidase substrate (luminol/4-iodophenol), and the light emission was measured using a luminometer (Berthold).

### Statistical analysis

All values are presented as means ± SEM. Student's paired *t*-test and one-way analysis of variance (ANOVA) with a post hoc Bonferroni test were applied to reveal the statistical significance of differences. Values of p < 0.05 were considered significant (*p < 0.05). Statistical analyses were performed using SPSS Software for Windows (Version 18; SPSS Inc., Chicago, IL).

## Results

### Survivin is expressed by human chondrocytes in osteoarthritis

**Figure 1:**
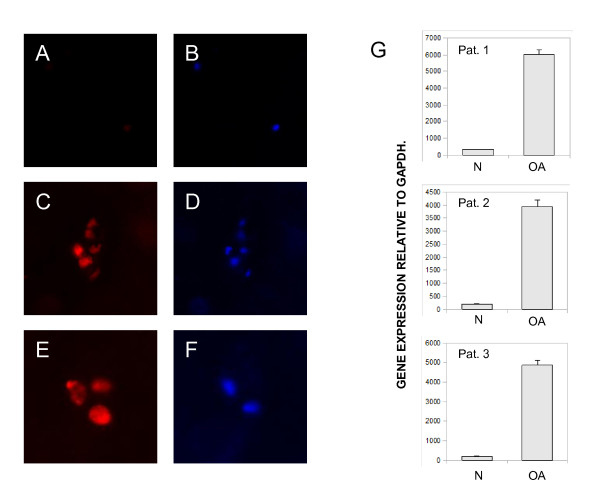
**Survivin expression in human paraffin-embedded osteoarthritic cartilage**. (A-F) Immunofluorescence for survivin (red) in macroscopically and microscopically arthritic cartilage (A, C, E) and adult non/moderate arthritic cartilage (paraffin-embedded tissue sections) (B, D, F). Chondrocytes were stained with 4,6-diamidino-2-phenylindole (blue) in the identical positions. Original magnifications: 200 × (A-D) and 400 × (E, F). (G) Cartilage from 10 patients was analyzed for the mRNA expression of survivin by semiquantitative real-time PCR. Histopathologic non/moderate arthritic cartilage samples (N) and osteoarthritic degenerated cartilage (OA) were analyzed by quantitative PCR. The relative gene expression rates of three representative patients are shown.

As a first step, we analyzed survivin expression in human osteoarthritic cartilage by immunofluorescence. We found no or only weak signals for survivin in macroscopically and microscopically non-arthritic cartilage, whereas survivin was readily detectable in osteoarthritic sections (Figure 1A-F). The strongest signals were seen in chondrocyte clusters in the deeper chondral layer, i.e. chondroid nests. The pattern of staining was predominantly nuclear or mixed cytoplasmic-nuclear. The cartilage matrix showed no survivin expression or autofluorescence. Next, we analyzed the survivin mRNA levels in osteoarthritic cartilage sections and macroscopically unaffected cartilage of the same joint. Real-time PCR from the non/moderate arthritic specimens showed very weak, if any, expression, whereas survivin expression was readily detectable in macroscopically arthritic cartilage (Figure 1G).

### Survivin is expressed at the protein and RNA levels in primary human chondrocytes

**Figure 2:**
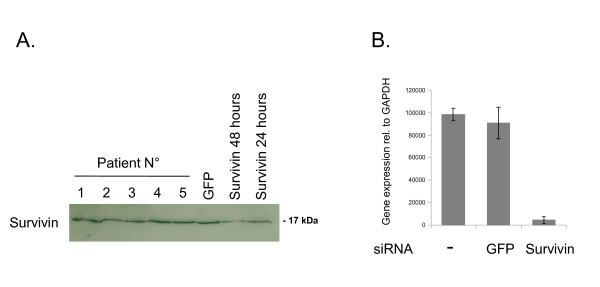
**Survivin expression in primary human chondrocytes**. (A) Immunoblot for survivin protein in five independent primary chondrocyte cultures (Patient No.). Protein lysates at 24 hours after transfection of a GFP siRNA and at 24 and 48 hours after transfection of a survivin-specific siRNA are shown. (B) Relative expression of survivin RNA after transfection of the specific siRNA detected by quantitative PCR. One representative chondrocyte culture is shown.

Survivin expression in primary human chondrocyte cultures was analyzed by immunoblotting and quantitative real-time PCR. Survivin protein was expressed in all cultures established at passage 2 (n = 5) as detected by immunoblotting (Figure 2A). Antibody specificity was confirmed by transfection of a survivin-specific siRNA, which led to a significant reduction in the detectable survivin protein after 24 hours (Figure 2A). Equal loading was controlled by β-actin detection and Coomassie brilliant blue staining of the membranes (data not shown). Survivin expression at the protein level showed a marked decrease at 48 hours after the siRNA transfection. Knockdown of GFP did not result in alterations of the survivin protein levels. Next, we analyzed survivin mRNA expression by applying quantitative real-time PCR. Survivin was detectable in all cultures analyzed (n = 4) and knockdown of survivin resulted in a marked reduction in detectable survivin RNA (Figure 2B).

### Subcellular survivin protein localization in primary human chondrocytes

**Figure 3:**
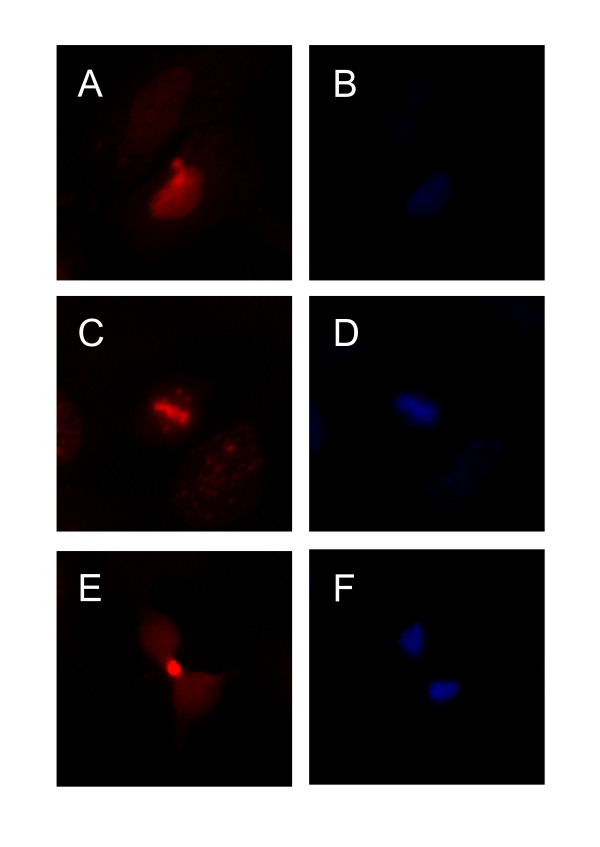
**Subcellular distribution of survivin protein in primary human chondrocytes**. (A-F) Immunofluorescence for survivin in primary chondrocytes cultured on glass slides (red). Staining with 4,6-diamidino-2-phenylindole (blue) of the identical positions is also shown. Survivin is located at the equatorial plate at metaphase in a mitotic cell (C, D). A midbody in a dividing cell with strong positive staining for survivin is shown (E, F).

We examined the subcellular survivin protein localization in primary human chondrocytes at passage 2 by immunofluorescence staining. Survivin was localized to heterogeneous subcellular compartments. Approximately 85% of cells revealed a predominantly cytoplasmic pattern of staining, while 12% had a mixed cytoplasmic-nuclear pattern and 3% had a purely nuclear pattern (Figure 3A and 3B). In sporadic cells, positive mitotic structures resembling a spindle apparatus and midbody could be detected (Figure 3C-F). Importantly, this pattern of staining was reported to be highly specific for immunocytological staining of survivin [29].

### Survivin knockdown leads to G2/M blockade, reduced rates of proliferation and sensitization of primary human chondrocyte cultures to proapoptotic stimuli

**Figure 4:**
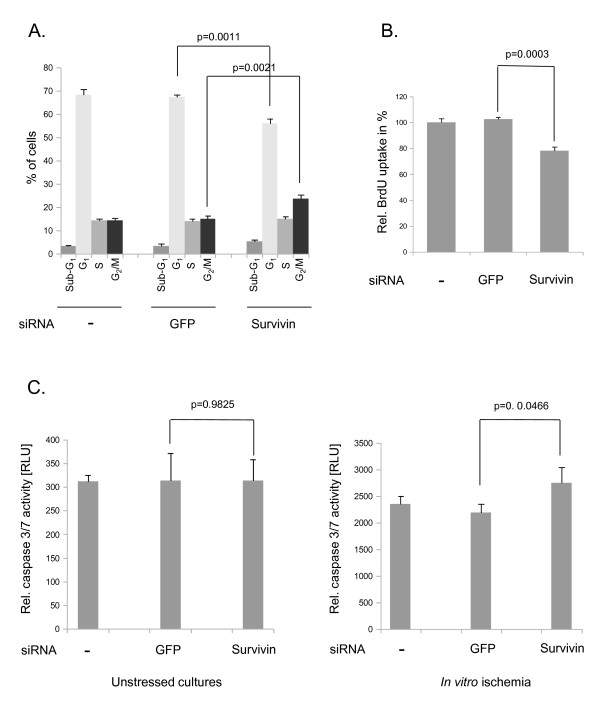
**Functional parameters after survivin knockdown in primary human chondrocytes**. (A) Cell cycle distribution at 48 hours after survivin knockdown (means ± SEM) as measured by the FACS/PI-staining method. GFP was used as a negative control. The original results of one representative experiment are shown. (B) BrdU uptake measured in relative light units at 48 hours after knockdown of survivin (means ± SEM). (C) Caspase activity at 24 hours after survivin knockdown in cells grown under regular culture conditions (unstressed) and under *in vitro *ischemia (1% oxygen, glucose deprivation) (means ± SEM). The results shown are a representative experiment of three independent experiments each performed in triplicate.

At 48 hours after knockdown of survivin by transfection of a specific siRNA, the cell cycle distribution was analyzed by the FACS PI method (Figure 4A). The siRNA transfection led to a significant reduction in the G1/0 fraction (68.4% to 56%) and marked elevation of the perimitotic G2/M cell phase fraction (14.3% to 23.77%). No significant alterations in the sub-G1 phase (p = 0.1478) and S phase (p = 0.2386) fractions were observed (Figure 4A). The effects of GFP transfection on the cell cycle distribution compared with untransfected cells were not significant (all p > 0.05). Next, we studied the effects of survivin knockdown on the proliferation of primary chondrocytes (Figure 4B). BrdU uptake was significantly (p = 0.0003) reduced at 48 hours after knockdown of survivin compared with GFP-transfected and untreated chondrocyte cultures (100%). The transfection of GFP led to no significant alterations in BrdU uptake after 48 hours compared with untransfected control cells (p = 0.3542). After studying the effects of the suppression of survivin on chondrocyte proliferation *in vitro*, we assayed the apoptotic activity at 24 hours after knockdown of survivin. In unstressed cultures, transfection of the survivin-specific siRNA did not lead to significantly altered caspase 3/7 activities (Figure 4C) (p = 0.9825). When exposed to *in vitro *ischemia (1% oxygen, glucose deprivation), caspase 3/7 activity increased significantly after transfection of the survivin-specific siRNA (Figure 4C) (p = 0.0466). Of note, when we compared the caspase 3/7 activity between untransfected and survivin siRNA-transfected cultures, this effect was not significant (p = 0.0678). ANOVA and a subsequent Bonferroni post hoc test revealed significantly increased apoptotic rates for all transfection conditions (untransfected + unstressed vs. untransfected + *in vitro *ischemia, p < 0.0001; GFP siRNA + unstressed vs. GFP siRNA + *in vitro *ischemia, p < 0.0001; survivin siRNA + unstressed vs. survivin siRNA + *in vitro *ischemia, p = 0.0002). The transfection of GFP had no significant influences on the apoptotic activity (all p > 0.05)

## Discussion

A finely orchestrated balance of chondrocyte proliferation and cell death enables endochondral ossification and subsequent skeletal growth. In adult cartilage, proliferation is limited to pathologic conditions, while constitutive prevention of apoptosis is a necessity to withstand stressors like mechanical forces, reactive oxygen species and cytokine exposure [6-8]. The antiapoptotic tumor gene survivin has been extensively studied in cell cycle and apoptosis assays in tumor cells, with little available data in primary cells and chondrocyte biology so far [30]. Previous studies stressed the "oncofetal" pattern of survivin gene expression and its absence in adult differentiated cells and tissues [9]. In recent publications, a role for survivin in RA has been discussed and convincing data about its importance for the progression of this inflammatory disease have been presented [18,24]. In the present study, we report on the expression of survivin at the protein and mRNA levels in human osteoarthritic cartilage. Here, the so-called chondroid nests, comprising accumulations of "stressed" chondrocytes, revealed a reactivation of survivin gene transcription and translation.

Furthermore, *in vitro *experiments with primary human chondrocytes suggested the possible functions of survivin in cartilage biology. Suppression of survivin gene expression by transfection of a specific siRNA resulted in marked alterations of the cell cycle distribution and inhibited G2/M progression. In addition, the proliferative activity of primary human chondrocytes decreased after specific knockdown of survivin, as measured by BrdU uptake. Importantly, we noted a discrepancy between the decrease in BrdU uptake at 48 hours and the lack of significant alterations in the S phase fraction at 48 hours after transfection of the survivin-specific siRNA. This might be explained by the unequal sensitivity and specificity of the applied assays [31]. Nevertheless, the marked increase in the G2/M cell phase fraction and the concurrent decrease in BrdU uptake underline the roles of survivin in cell cycle regulation and proliferation. A possible explanation of these effects could be a key function for the gene in the chromosomal passenger complex and a subsequent failure of mitotic cell division [32-34]. Interestingly, a recent study indicated that histone H3 phosphorylation is directly recognized by survivin before the activation of Aurora B takes place [35]. The second well-characterized function of survivin is the prevention of programmed cell death [36]. In our study, no significant alterations in the apoptotic activity in unstressed chondrocyte cultures were detected after knockdown of survivin. In contrast, after stressing the cells by *in vitro *ischemia, the knockdown of survivin resulted in elevated apoptotic rates. Interestingly, Gagarina et al. [30] reported on the upregulation of survivin and other IAP members by cartilage oligomeric matrix protein and subsequent cellular protection in primary chondrocytes. Of note, the authors of the study stressed the inducibility of the IAP family members, while the constitutive expression and function were not elucidated.

Considering the plethora of reports on inflammatory cytokines leading to an induction of survivin gene expression, no single relevant factor has been identified. Future studies need to dissect the interactions between proinflammatory mediators, mechanical influences and the resulting effects on survivin expression in human cartilage [37-39]. The interference with inflammatory pathways might modulate survivin function and gene expression [40]. Furthermore, new therapeutic attempts to directly suppress the apoptotic activity in osteoarthritis, could make use of the antiapoptotic capacity of survivin [41].

## Conclusions

In summary, we have demonstrated for the first time that the antiapoptotic protein survivin is re-expressed in osteoarthritic human cartilage and primary human chondrocytes, and our functional analyses indicated that survivin exerts both classic functions, i.e. cell cycle regulation and survival control. Learning more about survivin expression in chondrocytes might be an important step toward understanding cartilage biology and pathology, and could be of help in the development of future regenerative strategies.

## Competing interests

The authors declare that they have no competing interests.

## Authors' contributions

PL, JS and TR designed the research. PL, SB and TR performed the experimental study. PL and JS performed the statistical analyses. CB, SG and RHS participated in the data interpretation. PL, JS, RHS and JG drafted the manuscript. All authors read and approved the final manuscript.

## Pre-publication history

The pre-publication history for this paper can be accessed here:

http://www.biomedcentral.com/1471-2474/12/150/prepub
